# Supplementary material for: In-situ transfer vat photopolymerization for transparent microfluidic device fabrication
Source: Nat Commun. 2022 Feb 17;13:918. doi: 10.1038/s41467-022-28579-z (PMC8854570; doi:10.1038/s41467-022-28579-z)
Supplement: Supplementary file 1 — Supplementary Information [file 41467_2022_28579_MOESM1_ESM.pdf]

SUPPLEMENTARY INFORMATION FOR

In-situ Transfer Vat Photopolymerization for Transparent Microfluidic Device Fabrication

Yang Xu[1,2], Fangjie Qi[1,2], Huachao Mao[1,3], Songwei Li[1,4], Yizhen Zhu[1,4], Jingwen Gong[1,5], Lu Wang[5], Noah Malmstadt[5, 6], Yong Chen[1,2,4]*

*Corresponding author. Email: yongchen@usc.edu

[1]Center for Advanced Manufacturing, University of Southern California, Los Angeles, California 90007, USA.

[2]Daniel J. Epstein Department of Industrial and Systems Engineering, University of Southern California, Los Angeles, California 90089, USA.

[3]School of Engineering Technology, Purdue University, West Lafayette, Indiana 47907, USA.

[4]Department of Aerospace and Mechanical Engineering, University of Southern California, Los Angeles, California 90089, USA.

[5]Mork Family Department of Chemical Engineering and Materials Science, University of Southern California, Los Angeles, California 90089, USA.

[6]Department of Chemistry, University of Southern California, Los Angeles, California 90089, USA.

This document includes:

- Supplementary Figs. 1 to 9
- Supplementary Tables 1 to 7
- Supplementary Methods I to V
- Supplementary Discussion

Supplementary Figures

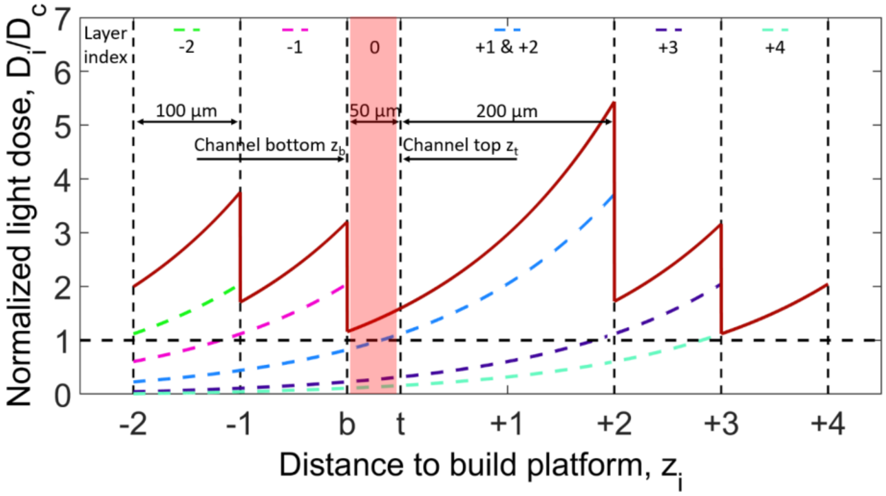

Supplementary Figure 1 | Normalized light dose distribution of each projection and the accumulated light dose along the z-direction when manufacturing the part in Fig. 1d via traditional VPP with increased channel roof thickness. Suppose the layer thickness is 100 μm and the curing depth is 125 μm. The channel roof thickness is 200 μm.

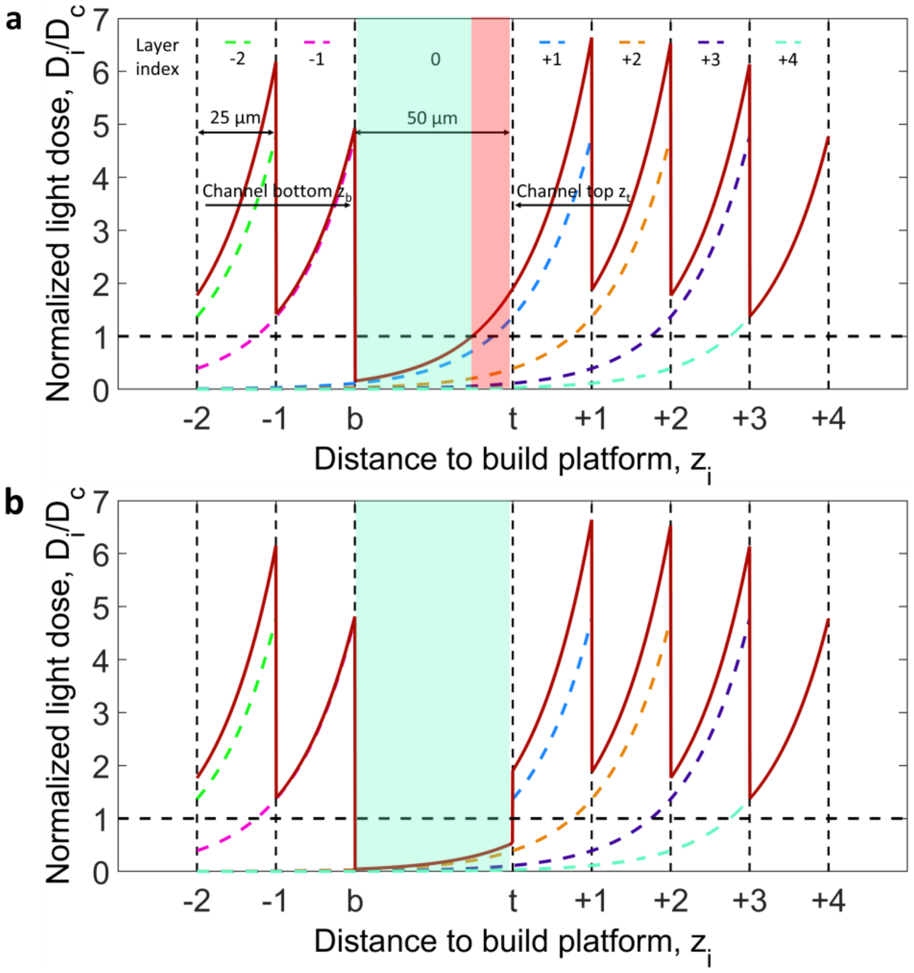

Supplementary Figure 2 | Comparison of the energy distribution when fabricating the part in Fig. 1d via conventional VPP (a) and our IsT-VPP (b) using materials with decreased light penetration depth. Suppose the layer thickness is 25 μm and the curing depth is 20 μm. The channel roof thickness is 25 μm.

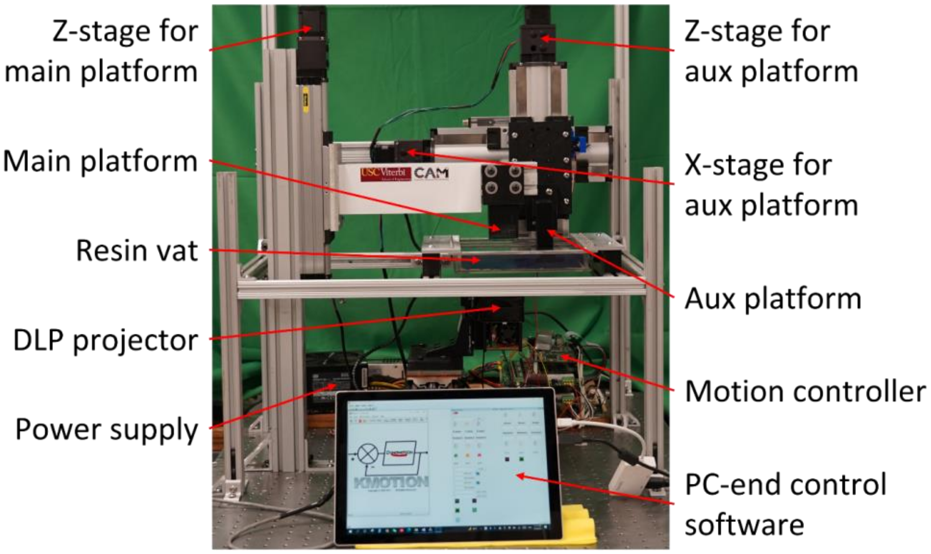

Supplementary Figure 3 | IsT-VPP experimental prototype.

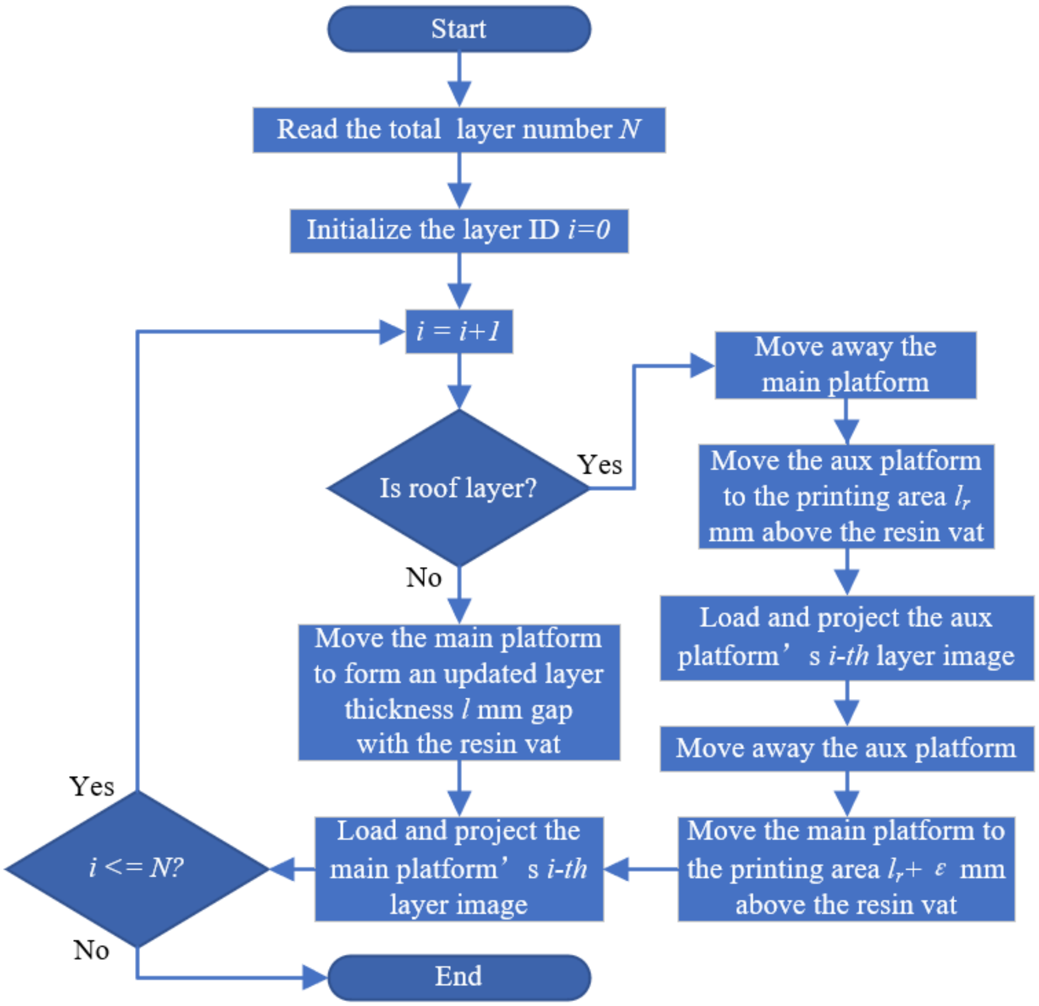

Supplementary Figure 4 | Printing process flowchart

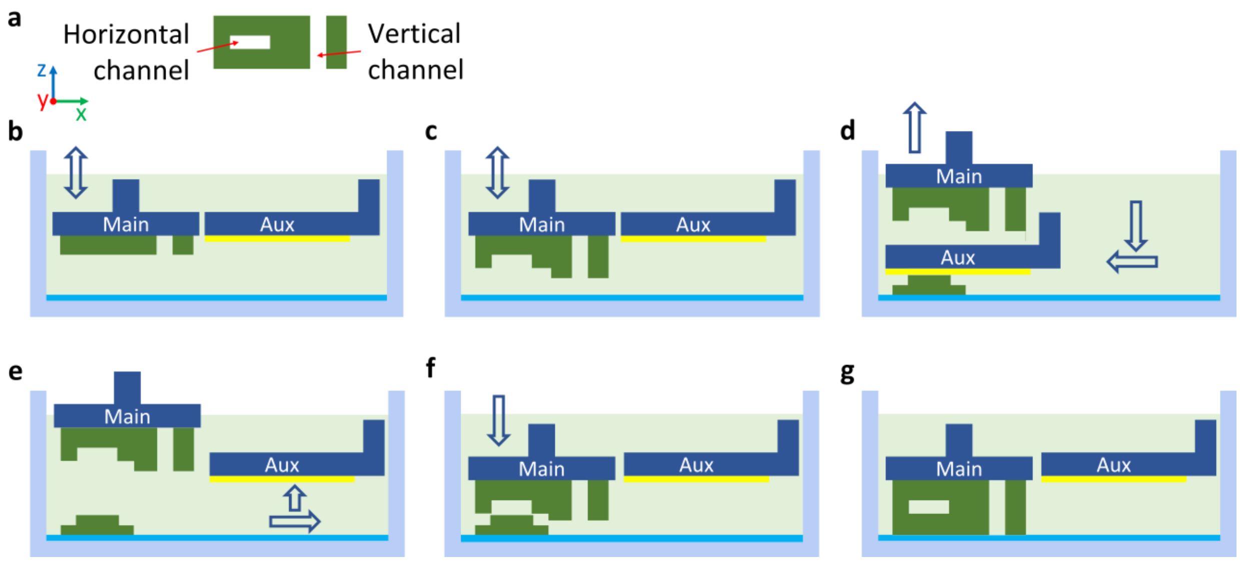

Supplementary Figure 5 | Printing process illustration for a part with both horizontal and vertical channels. (a) The cross-section view of the sample part. (b-g) Fabrication process. Note the sidewalls of the vertical channel can be fabricated using the regular layer thickness in step (c) without being affected by the Aux platform in step (d) or (e).

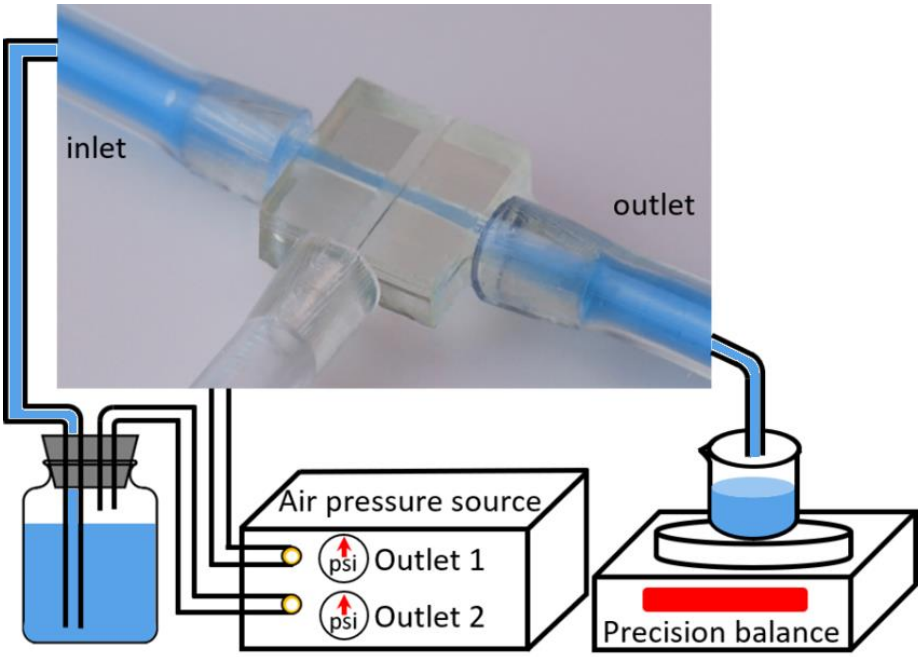

Supplementary Figure 6 | Setup for closing pressure test of the 3D-printed microfluidic valve. The flow channel inlet is connected to a bottle containing blue-dyed DI water. An electronically controlled air pressure source drives the liquid into the flow channel and provides air pressure to the control channel. The flow rate is obtained by measuring the mass of water flowing out of the outlet within 1 min.

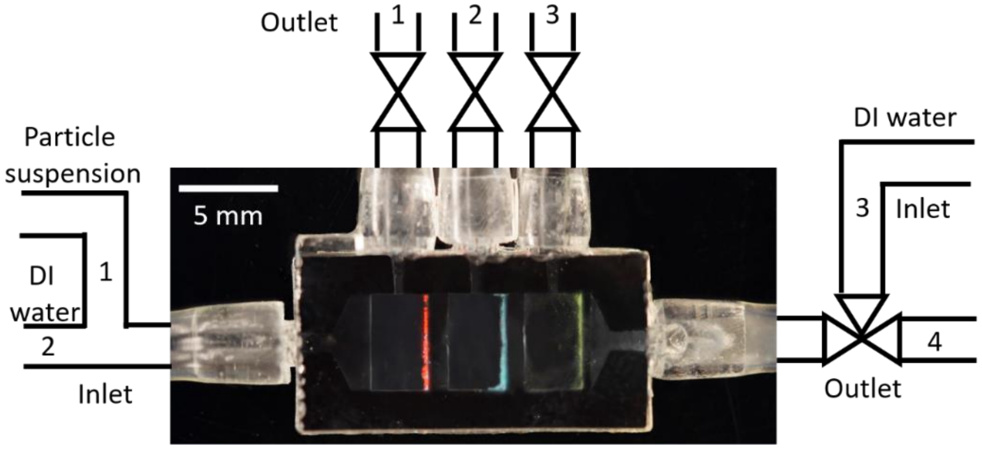

Sequence	Inlet 1	Inlet 2	Inlet 3	Outlet 1	Outlet 2	Outlet 3	Outlet 4	Flow rate (mL/min)
1	O	✖	✖	✖	✖	✖	O	10
2	✖	O	✖	✖	✖	✖	O	0.05
3	✖	O	O	✖	✖	O	✖	10
4	✖	O	O	✖	O	✖	✖	10
5	✖	O	O	O	✖	✖	✖	10

Supplementary Figure 7 | 3D-printed microparticle sorting system and the working sequences. In step 1, open outlet 4 and keep other outlets closed. Particle suspension flows into the filter chip through inlet 1. In step 2, DI water flows in and out of the chip via inlet 2 and outlet 4, pushing particles to each separator. In step 3, open outlet 3 and keep other outlets closed. DI water from inlet 2 and 3 with equal flow rate flush green particles out of outlet 3. In step 4, open outlet 2 and keep other outlets closed. DI water from inlet 2 and 3 flush blue particles out of outlet 2. In the last step, open outlet 1 and keep other outlets closed. DI water from inlet 2 and 3 flush red particles out of outlet 1.

Supplementary Tables

Supplementary Table 1 | Literature review on 3D-printed microfluidic channels and related technologies.

(**Note:** Entries are sorted according to the *z*-resolution, with those having the smallest channel height listed at the top. Our work can enhance VPP-printed microchannels' resolution without compromising transparency, size, and cross-sectional profile constraint. Other methods either suffer from sizes or cross-sectional profile constraints, or result in channels larger than 100 μm, or render the printed structures colored (Irgacure-819 and ITX are light yellow; NPS is yellow; Sudan I is deep orange) with significantly reduced fabrication speed.

PEG-DA: poly (ethylene glycol) diacrylate; Irgacure: phenylbis (2,4,6-trimethylbenzoyl) phosphine oxide; NPS: 2-nitrophenyl phenyl sulfide; TPU: thermoplastic polyurethane; LAP: lithium phenyl-2,4,6-trimethylbenzoylphosphinate; ITX: isopropyl thioxanthone.)

h_{min} (μm)	Part color	Materials	Light source	δ_p (μm)	Size or cross-sectional constraint	Process & apparatus	Ref
10	Transparent	Anycubic clear	405 nm LED	179	No	DLP-based VPP (customized)	**This work**
10	Transparent	Nanoscribe Photoresist IP-L 780	Femto-second laser		Yes	Micro molding and two-photon polymerization	42
18	Transparent yellow	PEG-DA-258 + 1% (w/w) Irgacure-819+ 3% w/w NPS	385 nm LED	8	No	DLP-based VPP (Visitech LRS-WQ)	39
40	Transparent	SainSmart Clear Flexible TPU			Yes	Material extrusion	24

						(Prusa i3 MK3 3D printer)	
54	Transparent	Stratasys VeroClear or MED610 (base material) + thin polycarbonate membrane (embedded physical barrier) + Stratasys Tango (cover material)			Yes	Material jetting (Stratasys J750)	30
60	Transparent deep orange	PEG-DA-258 + 1% (w/w) Irgacure-819+ 0.6% w/w Sudan I	405 nm LED	11	No	DLP-based VPP (Asiga Pico Plus 27)	35
100	Transparent yellow	PEG-DA-700 + 2.5% (w/w) LAP + 6% (w/w) Quinoline Yellow	365 nm LED		No	DLP-based VPP (TI DLP7000UV + Vialux Hi-Speed V-7000 + LED Engin LZ1-00UV00)	8
100	Transparent	Molten soda-lime glass			Yes	Material extrusion (MICRON3DP)	1
100	Transparent	acetoxy silicone Loctite SI 595 CL			Yes	Material extrusion (Nordson EFD Ultimus V)	23
200	Transparent	DSM Somos WaterShed XC 11122	354 nm laser		No	Laser-based VPP (3D Systems Viper SLA)	37
200	Transparent	Acrylonitrile (part material) + Wax (support material)			No	Material jetting (3D Systems ProJet 3500)	21

250	Transparent deep orange	PEG-DA-258 + 1% (w/w) Irgacure-819+ 0.2% w/w Sudan I	405 nm LED	~84	No	DLP-based VPP (B9Creator 3D printer v1.1)	36
300	Transparent deep red	EnvisionTEC R11	405 nm LED		No	DLP-based VPP (EnvisionTEC Perfactory P4 Mini)	34
500	Transparent	PEG-DA-258 + 0.4% (w/w) Irgacure-819 + 0.4% (w/w) ITX	385 nm LED	~144	No	DLP-based VPP (Asiga Pico2 HD 27)	41
500	Transparent	3D Systems VisiJet M3 (part material) and VisiJet S300 (support material)			No	Material jetting (3D Systems ProJet 3000HD+)	20
700	Transparent	PEG-DA-258 + 0.6% (w/w) Irgacure-819 + 0.6% (w/w) ITX	385 nm LED	~94	No	DLP-based VPP (Asiga Pico2-HD 27)	40

Supplementary Table 2 | Statistical result of 24 fabricated channel heights in Fig. 3e

**Target channel heights *H* (μm)**	**Fabricated channel height *h* (μm)**
10	$10.433^{+0.591}_{-0.984}$
20	$20.751^{+0.685}_{-0.491}$
30	$30.943^{+0.340}_{-1.022}$
40	$41.173^{+0.347}_{-0.441}$
50	$48.738^{+0.294}_{-0.293}$
60	$61.295^{+0.303}_{-0.484}$

Supplementary Table 3 | Printing parameters setting for all the demos in the main text

	USC-shaped router		Single-layer channels		Multiple-layer channels		Micro valve		Particle sorter
**Figure index**	1D		3A		3C		4E		5A
**Normal layer thickness l (μm)**	10	100	10	100	10	100	10	100	100
**Exposure time (s)**	0.45	0.85	0.45	0.85	0.45	0.85	0.45	0.85	0.85
**Roof layer thickness l_r (μm)**	200		200		65		25	200	200
**Exposure time (s)**	1.9		1.9		0.75		0.55	1.9	1.9
**Adhesive layer number**	4		4		4		4		4
**Exposure time for adhesive layers (s)**	4		4		4		4		4
**Gap size ε (μm)**	10		10		10		10		10
**Outward offset τ (pixel)**	4		4		4		4		4
**Energy reduction factor α**	0.6		0.6		0.6		0.6		0.6
**Energy reduction factor β**	0.5		0.5		0.5		0.5		0.5

Supplementary Methods

I. Measurement of light penetration depth.

To measure light penetration depth and determine exposure time for each layer, we measured the curing depth of different exposure times and generated the curing rate table as shown in Supplementary Table 4 and plotted in Fig. 1c. We applied the liquid resin on a glass slide, positioned it on the resin vat in the printer at the fixed light power, and cured it for 0.5s, 1s, 2s, 3s, 4s, 5s, and 6s, respectively. Then, gently remove the unreacted liquid resin from the glass slide with tissue paper and clean its surface with isopropanol. The thickness of the cured resin was measured using a digital microscope (Micro-Vu, Sol 161). Repeat this process ten times for each exposure time. The light penetration depth δ_p for a given resin can be derived by fitting the measured data with Jacob's working curve $c_d = \delta_p \ln\left(\frac{t_d}{T_c}\right)$. The curing rate table also provides essential guidance for choosing suitable exposure times.

Supplementary Table 4 | Curing rate table. The thickness of cured resin is presented vs. different exposure times (without aux platform).

**Exposure time (seconds)**	**Thickness of cured resin (μm)**
0.5	$52.4^{+7.6}_{-10.4}$
1	$132.8^{+10.2}_{-11.8}$
2	$264^{+9.0}_{-15.0}$
3	$332.4^{+7.6}_{-12.4}$
4	$410.8^{+14.2}_{-7.8}$
5	$453.8^{+7.2}_{-15.8}$
6	$481^{+14.0}_{-10.0}$

II. Experimental study on bonding force of different material interfaces.

We built a bottom-up-projection-based VPP 3D printer shown in Supplementary Fig. 8a to conduct the separation force study. Three force sensors (FlexiForce A201 Sensor, Tekscan, Inc., South Boston, MA) are sandwiched between the Z-stage frame and the build platform handle to measure the separation force in real-time. An Op-amp circuit (FlexiForce Quickstart Board, Tekscan, Inc., South Boston, MA) amplified and converted the sensor signal to voltage. Before the experiment, the sensors and the Op-amp circuits were calibrated according to the manufacturer's calibration procedure (Supplementary Fig. 8b-d). A linear function was used to fit the sensor readouts and the corresponding forces. The linear functions were utilized to measure the pulling-up forces during the separation process. An Arduino Uno microcontroller collected the voltage data through its analog-to-digital converter (ADC) inputs and then sent the readouts to a computer. The pulling force from the part was transferred to the sensors when the build platform was raised. Thus, the measured forces' sum reflected the separation process between the newly cured layers and the constrained vat surface, forming our analysis basis. The linear stage to move the build platform was driven by a KFLOP motion controller and a SnapAmp amplifier (Dynomotion Inc., Calabasas, CA). The 405 nm UV DLP projector served as the irradiation light source. A self-developed software system synchronized the whole experimental process, including sending commands to the microcontrollers and the DLP projector and acquiring data from the sensors.

The measuring procedure is similar to the typical bottom-up-projection-based VPP process (Supplementary Fig. 8e-g): (1) When the fabrication of one layer begins, the DLP projector projects a mask image to cure a thin layer of liquid resin. After a specific exposure time, the liquid resin is solidified between the previously cured layers and the vat surface, as shown in Supplementary Fig. 8e. (2) After the curing process, the computer begins to read and record the

force sensors' readouts until the end of the separation process (Supplementary Fig. 8f). Meanwhile, the build platform moves up Δz_1 mm at 0.1 mm/s to release the newly cured layer from the resin vat. (3) The build platform moves down Δz_2 mm to form a one-layer-thickness gap, as shown in Supplementary Fig. 8g. Now the setup is ready for printing the next layer (Supplementary Fig. 8e). The CAD model used in the experiments is given in Supplementary Fig. 8h. The pyramid-like model has four sections with different areas (15 mm × 15 mm, 25 mm × 25 mm, 35 mm × 35 mm, and 45 mm × 45 mm). The layer thickness l is 100 μm. There are 40 layers for the base section and 20 layers for each of the other three sections. The separation force measurement for each parameter set was repeated 20 times. To avoid the influence from surface tension, we only use the data collected from each section's last 10 layers for analysis. The base section's extra 20 layers eliminated the influence of the tolerance between the build platform starting position and the resin vat in the first several layers.

We measured the separation forces of different material interfaces by switching the resin vat's coating materials (PDMS, FEP film, or none) and changing the build platform's cover (glass or plate printed by the same resin). The recorded pulling force against the separation time is shown in Supplementary Fig. 8i-l. The peak values derived from the curves are the ultimate separation forces. They are summarized and plotted in Fig. 2b. The separation force required to detach the polymer-FEP interface is always larger than the polymer-PDMS interface, given the same contact area from the curves.

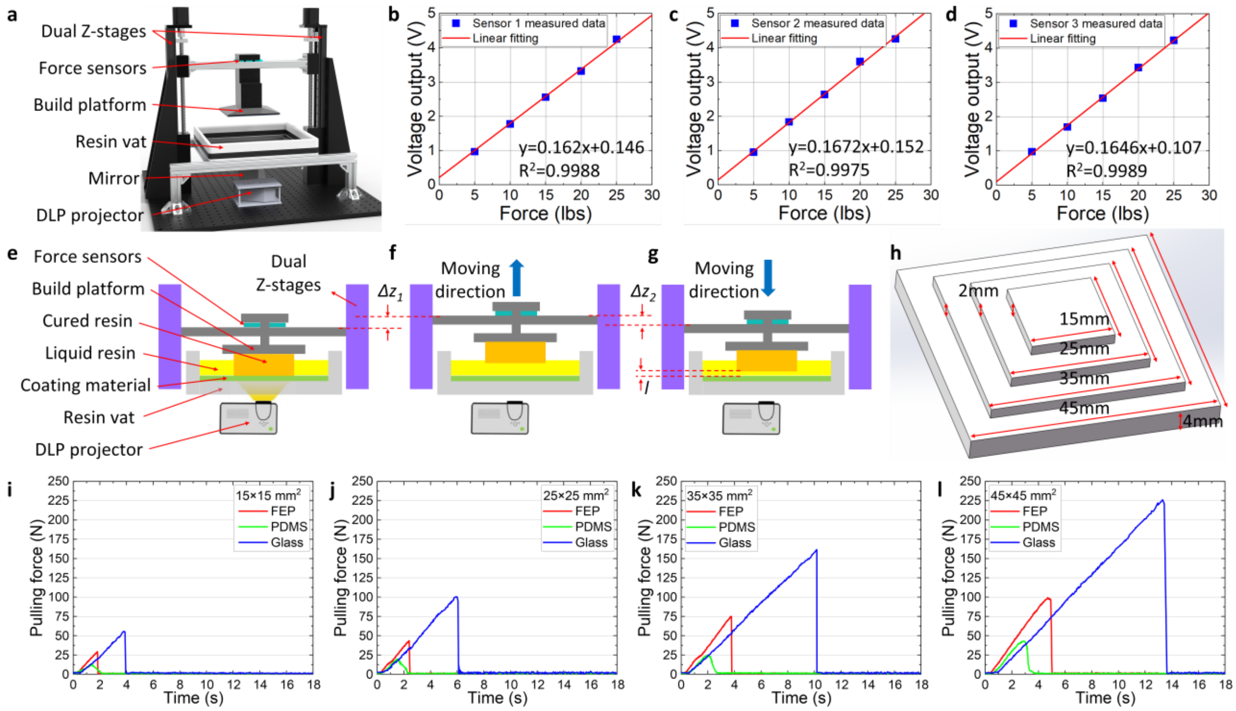

Supplementary Figure 8 | Experimental study on bonding force of different material interfaces. (**a**) The setup used to test bonding forces. The top portion of the build platform is cut open for an easy view of the force sensors. The resin vat was coated with different materials (PDMS or FEP film) or just naked glass with no coating. The build platform was either covered by a glass or a 3D-printed plate using the same resin. Both of them were connected to the aluminum frame via construction glass glue. (**b-d**) Calibration results of the force sensors. (**e-g**) Separation force measurement procedure. (**e**) Project a mask image to cure the liquid resin. (**f**) Move up the build platform by a small distance Δz_1 mm to release the cured layer from the resin vat. The sensor readouts are collected and recorded through the whole moving-up process. (**g**) Move down the build platform by Δz_2 mm to form a one-layer-thickness gap l. (**h**) CAD model used in the experiments. The cross-sectional areas of the pyramid-like model are $15\times15\text{mm}^2$, $25\times25\text{mm}^2$, $35\times35\text{mm}^2$ and $45\times45\text{mm}^2$, respectively. The designed layer thickness l is 100 µm. There were 40 layers for the base section and 20 layers for each of the other three sections. (**i-l**) Pulling force

records for various material interfaces and contact areas during the separation procedure. (**i**) $15\times15\text{mm}^2$. (**j**) $25\times25\text{mm}^2$. (**k**) $35\times35\text{mm}^2$. (**l**) $45\times45\text{mm}^2$.

III. Analytical model.

The critical light dose can be expressed by

$$D_c = t_i I_i e^{-c_i/\delta_p} \tag{1}$$

where c_i is curing depth for layer i. By substituting Supplementary Equation (1) into Equation (1), the light dose delivered to the channel top is

$$D_t = D_c \sum_{i=1}^{n} e^{\frac{c_i-(z_i-z_t)}{\delta_p}} \tag{2}$$

Suppose we use uniform layer thickness l and curing depth c_d to print all the after-roof layers. Since there is no energy delivered to the channel when curing the channel roof in the proposed approach, then

$$D_t = D_c \sum_{i=2}^{n} e^{\frac{c_d-[l_r+(i\text{-}1)l]}{\delta_p}} = D_c \frac{e^{(c_d-l_r-l)/\delta_p}(1-e^{-(n\text{-}1)l/\delta_p})}{1-e^{-l/\delta_p}} \leq D_c \tag{3}$$

The curing depth is usually in the range of 1.1 to 1.5l. Suppose c_d is set to 1.25l and set n to $+\infty$, then we can get a critical value for the roof thickness l_r

$$e^{(c_d-l_r-l)/\delta_p} + e^{-l/\delta_p} \leq 1 \Leftrightarrow l_r \geq 0.25l - \delta_p \ln(1-e^{-l/\delta_p}) \tag{4}$$

The critical value (177 μm in our case) is a threshold, meaning the energy coming from the subsequent exposures can be ignored after fabricating a l_r-mm thick channel roof using the aux platform (the bottom half of Fig. 1e and Supplementary Fig. 2b). Otherwise, a grayscale mask image is required for the channel portions. From Supplementary Equation (4), critical roof

thickness l_r will increase for larger δ_p. By contrast, the accumulated light dose at channel top D_t' by traditional VPP will always exceed critical dosage D_c, as shown in Supplementary Equation (5), the top half of Fig. 1e, Supplementary Fig. 1, and Supplementary Fig. 2a.

$$\begin{aligned} D_t' &= D_c \sum_{i=1}^{n} e^{\frac{c_i-(z_i-z_t)}{\delta_p}} = D_c \sum_{i=1}^{n} e^{\frac{c_d-il}{\delta_p}} \\ &= D_c \frac{e^{(c_d-l)/\delta_p}(1-e^{-nl/\delta_p})}{1-e^{-l/\delta_p}} = D_c \frac{e^{c_d/\delta_p}-e^{(c_d-nl)/\delta_p}}{e^{l/\delta_p}-1} > D_c \end{aligned} \tag{5}$$

IV. Mask image planning.

To make the presented IsT-VPP universally applicable to general microfluidic applications, we also developed an image planning algorithm to handle complex channel networks. The algorithm is summarized in Supplementary Fig. 9 and Supplementary Table 4.

First, the input 3D CAD model represented by a .stl file is sliced into N 2D images $\{L_i^0\}_{i=1}^{N}$ as a basis according to a given layer thickness l. Each 2D image is a matrix with the same dimension as the projector's pixel resolution (e.g., 1280×800). Each matrix element is an integer value, ranging from 0 to 255, representing the duty ratio of each microscopic mirror's on/off state in the digital micromirror device (DMD).

Second, we have calculated the critical value for the roof thickness l_r given a resin type in the analytical model section. The critical roof thickness l_r is further converted to a critical layer number *k*, the number of layers affected by current exposure with the highest grayscale level 255.

Third, for each layer containing a channel roof L_i^0, a mask image is created for the main build platform L_i^1 and its counterpart L_i^2 for printing the channel roof portion via the aux platform.

Both images are initialized L_i^0. The channel area is defined as $L_i^0 - L_{i\text{-}1}^0$. The corresponding roof area is derived by offsetting the channel area outward with τ pixels. In the mask image L_i^2 , the grayscale level in the non-roof area is decreased by an energy reduction factor $0 < \alpha < 1$ (corresponding to Fig. 2f). In the mask image L_i^1, which is used to connect the channel roof with the previously built part, the channel area is set to 0 to avoid the over-curing issue (corresponding to Fig. 2h). For the void area within the previous k layers that may be affected by exposure L_i^1, the grayscale level in such places is reduced by an energy reduction factor $0 < \beta < 1$.

Finally, generate G-code for the main and aux build platforms. If the following $k-1$ layers are identical to the current roof layer, change the layer thickness to $k \times l$, and update the layer index (Fig. 1f-h). Otherwise, use the original layer thickness l (Fig. 3c and d). The G-code for the previous layer L_{i-1}^1 also needs to be adjusted accordingly. If the channel height h is larger than the gap size ε, change the L_{i-1}^1 layer thickness to $l - \varepsilon$ (corresponding to Fig. 2e). Otherwise, delete the layer L_{i-1}^1 and skip step 2 in Fig. 2e (Fig. 3b). This process is described in Supplementary Table 4 Algorithm 2.

For non-roof layers, initialize L_i^1 with the original mask image L_i^0. Similarly, the void area of previous k layers in L_i^1 will be modified. G-code for non-roof layers was generated as usual. In our work, we utilized in-house-developed software for slicing 3D models and contour offsetting[1, 2]. All the algorithms were realized in C++.

Supplementary Table 5 | General mask image planning algorithm for 3D printing microfluidic chips via IsT-VPP.

**Algorithm 1:**	Generate mask images and G-code
**Input**:	3D CAD model *.stl*, layer thickness l, critical roof thickness l_r, minimum gap ε, outward offset τ , and energy reduction factor α , β
**Output**:	Mask images L_i^1 and L_i^2 for the main and aux build platforms of each layer i, *G-code*

$\{L_i^0\}_{i=1}^N \leftarrow$ sliceTo2D(*.stl*, l);
$k \leftarrow \lceil l_r / l \rceil$;
for $i \leftarrow 1$ **to** N **do**
    if isRoof (L_i^0) **then**
        $L_i^1 \leftarrow L_i^0$; $L_i^2 \leftarrow L_i^0$;
        $channelArea \leftarrow L_i^0 - L_{i-1}^0$;
        $roofArea \leftarrow$ offset($channelArea, \tau$);
        L_i^2[**NOT** $roofArea] \leftarrow 255 \times \alpha$;
        $L_i^1[channelArea] \leftarrow 0$;
        $L_i^1[L_{i-1}^0 == 0 \bigcup L_{i-2}^0 == 0 \bigcup \cdots \bigcup L_{i-k-1}^0 == 0] \leftarrow 255 \times \beta$;
        $i \leftarrow$updateGCode($\{L_i^0\}_{i=1}^N$, i, l, l_r, ε, β);
    else
        $L_i^1 \leftarrow L_i^0$;
        $L_i^1[L_{i-1}^0 == 0 \bigcup L_{i-2}^0 == 0 \bigcup \cdots \bigcup L_{i-k-1}^0 == 0] \leftarrow 255 \times \beta$;
        generateGCode(i, l, $roofLayer$ = **false**);
return $\{L_i^1\}_{i=1}^N$, $\{L_i^2\}_{i=1}^N$, *G-code*;

**Algorithm 2:**	Generate G-code for roof printing and modify G-code for the previous layer: updateGCode($\{L_i^0\}_{i=1}^N$, i, l, l_r, ε, β)
**Input**:	Initial sliced 2D images $\{L_i^0\}_{i=1}^N$, current layer index i, layer thickness l, roof thickness l_r, minimum gap ε, and energy reduction factor β
**Output**:	Next layer index i

if channelHeight(i) > ε **then**
    generateGCode ($i-1$, $l-\varepsilon$, $roofLayer$ = **false**);
else

deleteLayer($i-1$);

$k \leftarrow \lceil l_r / l \rceil$;

if $L_i^0 = L_{i+1}^0 = \cdots = L_{i+k-1}^0$ **then**

generateGCode (i, $k \times l$, *roofLayer* = **true**);

$i \leftarrow i + k - 1$;

else

generateGCode (i, l, *roofLayer* = **true**);

return i;

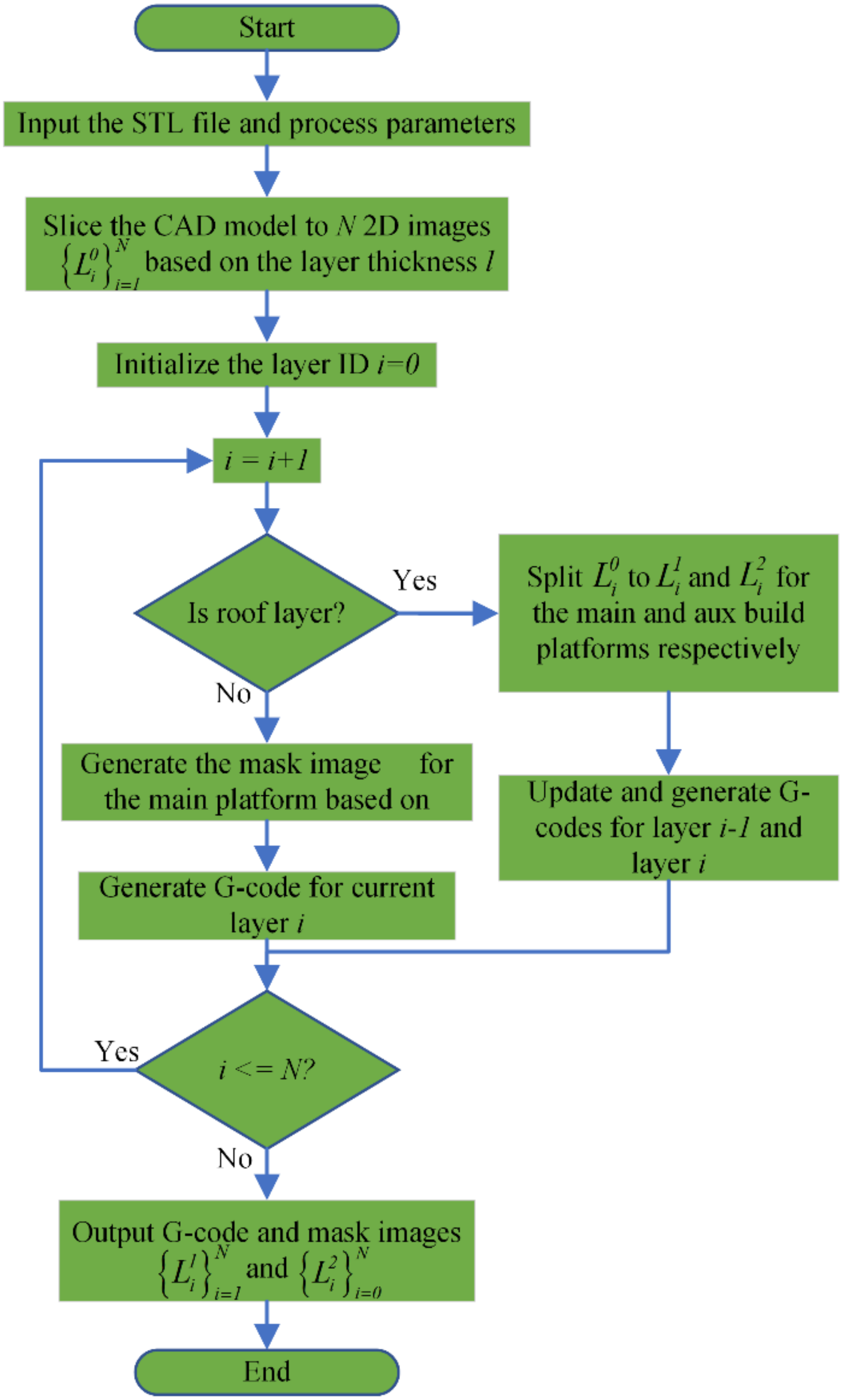

Supplementary Figure 9 | The pipeline of the mask image planning algorithm.

V. Guidelines of IsT-VPP setup based on common DLP/LCD 3D printers.

The presented IsT-VPP approach can be applied to commercial DLP/LCD resin 3D printers by extending the existing hardware and software systems. Here we gave some basic guidelines for future researchers and technicians. The recommended hardware and software are listed in Supplementary Table 6.

Supplementary Table 6 | Recommended hardware and software for IsT-VPP based on common DLP/LCD 3D printers.

**Hardware**	Motorized linear stage × 2
	Microcontroller board × 1
	Stepper motor driver with two outputs × 1
	Photoelectric limit switch × 1
	Print platform × 1
**Software**	Printing file preparation software for DLP/LCD resin printers

Additional two linear stages used to move the aux build platform in both *X* and *Z* axes can be placed right beside the commercial resin 3D printer. An external microcontroller is used to monitor the signal from the photoelectric limit switch mounted on the top of the *Z*-stage. Once the main build platform hits the limit switch, the microcontroller drives the motorized linear stages to move the aux build platform to form a layer thickness gap with the resin tank. After the first exposure for the roof layer, the aux build platform returns to its home position. Then the *Z* stage moves the main build platform down to prepare for the second exposure. After the two exposures, the channel is successfully fabricated. The non-roof layers are manufactured via the normal printing process using only the main build platform. To coordinate the movement of each

component, the printing file software system needs to be modified so the printing job file can be generated according to the presented IsT-VPP building process.

VI. Supplementary discussion

Staircase effect on the 3D printed microvalve performance.

Due to the staircase effect, there will be residual liquid after closing the valve in the gap between the valve membrane and valve seat at the region highlighted by yellow dash lines in the inset of Fig. 4m. However, based on the design, this gap should be smaller than the layer thickness (10 μm used in our case, see Supplementary Table 3 for details) – suppose the average gap is 5 μm. Given the following parameters (see Supplementary Table 7), we can estimate the gap effect based on the Darcy-Weisbach Equation $\frac{\Delta p}{L} = \frac{128}{\pi}\frac{\mu Q}{D^4}$. The flow rate Q will be as low as 118×10^{-5} μl/s, which can be ignored. This is a conservative estimation since we have not considered the pressure drop caused by the length of the channel and the plastic hose.

Supplementary Table 7 | Parameters used to estimate the staircase effect on valve performance.

Pressure drop Δp	3 psi
Valve seat length L	300 μm
Dynamic viscosity of water μ	8.90×10^{-4} Pa·s
Hydraulic diameter D	5 μm

References

1. Huang, P., Wang, C. C. L. & Chen, Y. Intersection-free and topologically faithful slicing of implicit solid. *J. Comput. Inf. Sci. Eng.* **13**, 1–13 (2013).
2. Chen, Y. & Wang, C. C. L. Uniform offsetting of polygonal model based on Layered Depth-Normal Images. *CAD Comput. Aided Des.* **43**, 31–46 (2011).
