# Supplementary material for: In-situ transfer vat photopolymerization for transparent microfluidic device fabrication
Source: Nat Commun. 2022 Feb 17;13:918. doi: 10.1038/s41467-022-28579-z (PMC8854570; doi:10.1038/s41467-022-28579-z)
Supplement: Supplementary file 3 — Description of Additional Supplementary Files [file 41467_2022_28579_MOESM3_ESM.pdf]

Description of Additional Supplementary Files

File Name: Supplementary Movie 1
Description: Simulation of IsT-VPP process

File Name: Supplementary Movie 2
Description: 3D printing microfluidic channels via IsT-VPP process

File Name: Supplementary Movie 3
Description: Demo of 3D-printed microfluidic valve

File Name: Supplementary Movie 4
Description: Demo of 3D-printed specimen platform

File Name: Supplementary Movie 5
Description: Demo of 3D-printed microparticle sorting device
